# Psycho-Behavioral Profiles of Pediatric Inpatients with Past and Recent Onset of Nonsuicidal Self-Injury: A Cluster Analysis Approach

**DOI:** 10.3390/jcm11154602

**Published:** 2022-08-07

**Authors:** Alessia Raffagnato, Sara Iannattone, Rachele Fasolato, Maria Paola Rossaro, Andrea Spoto, Michela Gatta

**Affiliations:** 1 Children and Adolescents Neuropsychiatry Unit, Woman and Child’s Health Department, Padova University Hospital, 35128 Padova, Italy; 2 Department of General Psychology, University of Padova, 35131 Padova, Italy

**Keywords:** nonsuicidal self-injury, inpatients, adolescence, maintenance factors, risk factors, cluster analysis

## Abstract

Few studies have focused on the persistence of nonsuicidal self-injury (NSSI) over time in developmental age. This study aimed to define the psycho-behavioral profiles of young inpatients according to past or recent NSSI onset (i.e., NSSI for more or less than one year, respectively), and identify possible risk factors for maintaining NSSI over time. A total of 118 Italian NSSI inpatients aged 9–17 were involved. The Youth Self-Report (YSR) was administered. K-means cluster analyses were conducted using the YSR affective disorders, social competencies, and social problems scales as clustering variables. A binomial logistic regression was run to clarify which of these variables discriminate between the past and recent NSSI onset groups. Chi-square tests were performed to pinpoint the variables associated with long-standing NSSI. The final cluster solution displayed four psycho-behavioral profiles; a greater number of inpatients with recent NSSI onset was found in the clusters characterized by scarce social competencies. Affective disorders and social competencies were significant predictors, and higher scores on both scales were more likely in the past NSSI onset group. School problems and alcohol/substance use were related to long-standing NSSI. Therefore, a lack of social skills may be involved in recent NSSI onset, while affective disorders and other problem behaviors may dictate the continuation of NSSI over time.

## 1. Introduction

Self-injurious behaviors—enacted intentionally and with the awareness of causing damage to oneself [1]—can be divided into suicidal and nonsuicidal on the basis of the desire to procure death with said behavior [2,3]. Nonsuicidal self-injury (NSSI) is very common among adolescents. In fact, adolescence represents a vulnerable period for the onset of NSSI: its prevalence reaches a peak at 15–16 years of age [4] and then decreases in adulthood [5,6]. Some works have shown an NSSI prevalence between 7.5% and 46.5% in the general adolescent population [7], and between 30% and 61% in psychiatric patients [8,9]. In the Italian context, some studies have pointed out a prevalence of NSSI in teenagers of between 13% and 42% [10,11,12]. Moreover, a progressive increase in adolescent NSSI had already been recorded worldwide before the COVID-19 pandemic, but especially in the last two years [7,13].

The personal and clinical features of young patients with NSSI include psychiatric familiarity [14], borderline personality functioning [15,16], traumatic events [8,17], history of bullying [18], school problems [19], alcohol and substance abuse [20], and problems in the family context [21]; this, when studied with the specific observational tools of family interaction, resulted in being characterized by the absence of emotional validation [22,23,24].

The literature reports different evolutionary trajectories of NSSI in adolescents, differentiating NSSI as a transient phenomenon or as a chronic and stable symptom [25,26]. Most studies have focused on NSSI frequency, distinguishing between habitual and episodical NSSI. However, together with frequency, another aspect to be examined is the NSSI course in terms of temporal duration (i.e., considering the time elapsed from onset to the last self-injurious act). In these terms, the majority of longitudinal works have found a chronic course of NSSI in adolescence [27,28,29,30]. Furthermore, a past history of NSSI has been confirmed to be the most important predictive factor for future NSSI [31,32].

The mechanisms underlying the stabilization and chronicization of NSSI are based on positive and negative self-reinforcement, increased pain tolerance (which facilitates the self-injurious act), and implicit identification with a “self-injurious identity” [2,28]. After a self-injurious act, the individual may experience emotional relief, which reinforces the use of NSSI as a stable behavior, useful for avoiding negative emotional states [33]. Other short-term effects reinforcing NSSI are the increase in positive emotions, a reduction in intrapersonal difficulties, and growing attention and help received from an interpersonal context [34]. A relevant factor in the long-term maintenance of NSSI is emotional dysregulation [35,36,37]. In particular, people who continue to engage in NSSI show a higher use of emotion suppression and a higher fatality rate with self-injurious acts [35]. Regarding this last point, the presence of NSSI is a well-established risk factor for suicidal ideation and behaviors [38,39,40].

The international literature reports both intrapersonal and interpersonal factors in association with the onset and maintenance of NSSI [2,41]. Nock [42], in his integrated model, associated the onset of NSSI with proximal factors, such as the presence of intense negative emotions, low stress tolerance, and difficulties in communication and problem solving; such factors were, in turn, linked to distal factors, such as child abuse, genetic predisposition, high emotional reactivity, and family hostility. The author stated that the maintenance of NSSI over time, differently from its onset, is influenced by processes of affective regulation and its consequences on the surrounding social context [3]. Subsequent evidence has confirmed these results, identifying a stronger association between the maintenance of NSSI over time and its intrapersonal function [30,41,43].

To date, only a few works have compared patients with recent versus past onset of NSSI. One of these was conducted by Victor et al. [30], who, by comparing adolescents and young adults (mean age: 16.1 years) with a history of NSSI lasting less or more than one year, evidenced that those with a longer NSSI history were older and mainly used NSSI with an intrapersonal function (e.g., affect regulation). Conversely, an interpersonal function of NSSI (e.g., obtaining a positive or negative reinforce from the social context) did not seem to be associated with the duration of NSSI. These findings highlighted the predominance of an intrapersonal function in patients with more chronic NSSI (i.e., NSSI lasting more than 12 months), although the frequency of NSSI over the past year, which can be considered a measure of its severity, may play a role in the association between function and duration of NSSI. Emery et al. [44], by conducting a longitudinal study on a sample of 730 adolescents (mean age: 13.4 years), took into consideration the presence of NSSI at two different time points in patients’ history; they found a lower satisfaction of psychological needs (autonomy, competence and relatedness) in the “maintenance” group (NSSI at both time points) compared to the “onset” group (NSSI only in the second time point) and the “cessation” group (NSSI only in the first time point), but a statistically significant difference emerged with the control group (no history of NSSI) only. The authors highlighted that both the “onset” and “cessation” groups were in a transitional period in which needs satisfaction had just begun to change, so additional time would have been necessary to observe the effect of the group membership.

### The Current Study

As highlighted above, there is still a paucity of studies specifically focusing on the clinical and personal factors linked to the maintenance of NSSI behavior over time in adolescence. Given the worldwide increasing prevalence of NSSI in adolescents and preadolescents [13], the present research aimed to fill the gaps in the literature on this topic, in order to identify in advance youth at risk of persistent NSSI and to implement prompt interventions.

In particular, the purpose of this study was twofold:

(a) To define the clinical and psycho-behavioral profiles of inpatients according to the onset of NSSI (i.e., past vs. recent). Specifically, since previous works have highlighted the relevance of emotion regulation function in maintaining NSSI behavior over time [30], we expected inpatients with past NSSI onset (i.e., NSSI for more than one year at the time of hospitalization) to present less interpersonal difficulties and more affective problems compared to inpatients with recent NSSI onset (i.e., NSSI for less than one year).
(b) To explore which personal and clinical variables may constitute risk factors for the maintenance of NSSI behavior over time. We considered some of the main factors previously found to be linked to nonsuicidal self-harming behavior per se, namely, bullying [18], school problems [19], family problems [21], psychiatric familiarity [14], alcohol and substance abuse [20], traumatic life events [17], and borderline intrapsychic functioning [15]. Given that, to the best of our knowledge, no research has investigated the role of these variables in the specific comparison between recent and past NSSI onset yet, we did not formulate any specific hypothesis.

## 2. Materials and Methods

### 2.1. Participants

The sample was composed of young inpatients with a history of NSSI who attended a neuropsychiatry unit in northern Italy between January 2013 and December 2019. For all participants, it was the first admission when the history of NSSI emerged.

To be specific, the sample as a whole included 118 NSSI inpatients: 102 girls and 16 boys, from 9 to 17 years of age (M = 14.4, SD = 1.59). The average age of the onset of NSSI was 13.1 years (SD = 1.39). We considered the following exclusion criteria: diagnosis of a neurodevelopmental disorder and the absence of NSSI in the previous 12 months.

Subsequently, the whole sample was divided into two groups according to the onset of NSSI: the first group, called “recent NSSI onset”, was composed of inpatients with a brief history of NSSI (i.e., NSSI behavior for less than 12 months at the time of hospitalization), while the second group, labeled “past NSSI onset”, consisted of inpatients with a long history of NSSI (i.e., NSSI behavior for more than 12 months). The sociodemographic and clinical characteristics of the two groups are reported in the Results section.

### 2.2. Procedure

The current research is an observational retrospective study based on the data collected from the patients’ paper-based and computerized clinical records. In particular, we considered anamnestic data about family, schooling, peer relationships, substance and alcohol use, traumatic life events, chronic pathologies, eating problems, previous hospitalizations, and admissions to other mental health services. We also examined the reason, access mode, and duration of the current hospitalization, ICD-10 [45] diagnosis, ongoing pharmacological and psychological treatment, post-discharge services, and intrapsychic functioning (i.e., neurotic, borderline, and psychotic) defined on the basis of Kernberg’s criteria [46]. Moreover, we collected information about nonsuicidal self-injurious acts (frequency, functions, number of self-injured body parts, and age of the onset) and suicidal phenomena (suicidal ideation, suicide attempts, and suicidal method). Finally, we analyzed the scores on the psychodiagnostic tests administered to inpatients during clinical interviews for their diagnostic assessment (see below).

The recruitment was submitted to the informed consent of inpatients and their parents. Moreover, the research was conducted in accordance with the Declaration of Helsinki and approved by the Institutional Ethics Committee of the local University Hospital.

### 2.3. Measures

The standardized tool considered in the current study was the Youth Self-Report (YSR; [47]; Italian version: [48,49]). This is among the scales most often used in clinical settings and research for the assessment of juvenile behavior. It yields two profiles: one regarding competences (activities, social functioning, and school performance), while the other concerns behavioral and emotional problems. These can be classified as “normal,” “borderline,” or “clinical” on eight syndrome scales, which are anxiety/depression, withdrawal, somatization, social problems, thought-related problems, attention problems, and aggressive and rule-breaking behavior. Such scales can be grouped into three broad-band scales: internalizing problems, externalizing problems, and total problems. In addition, there are six DSM-oriented scales: affective problems, anxiety problems, somatic problems, hyperactivity/attention deficit, oppositional–defiant problems, and conduct problems. Regarding the internal consistency of the instrument, Frigerio and colleagues [48] found Cronbach’s α values ranging from 0.83 to 0.91. In the present study, Cronbach’s alpha coefficients for each scale ranged from 0.77 to 0.93. Finally, the Deficient Emotional Self-Regulation (DESR) Profile can be calculated by summing the scores on the attention problems, anxious/depressed, and aggressive problems scales. The DESR Profile is considered an index of behavioral and emotional dysregulation. A score between 180 and 210 indicates deficient emotional self-regulation, while a score equal to or higher than 210 indicates severe dysregulation [50,51].

### 2.4. Statistical Analysis

In the preliminary analyses, descriptive statistics and frequency tables were calculated to outline the sociodemographic and clinical features of both the whole sample and the two subgroups of inpatients with past and recent NSSI onset. Independent sample t-test and a chi-square (**χ**^2^) test were also run to check for the absence of significant differences between the two subgroups in terms of age and sex, respectively.

The subsequent statistical analyses can be divided into two steps based on the afor**e**mentioned objectives: the first one concerned the delineation of the psycho-behavioral profiles of patients according to the onset of NSSI (i.e., past vs. recent); the second one focused on investigating whether some of the factors thought to underlie NSSI were specifically related to its past onset, thus representing risk factors for the maintenance of nonsuicidal self-injurious behavior over time.

Pertaining to the first step, a cluster analysis procedure was applied. Specifically, a series of k-means cluster analyses were conducted, using the social problems, social competencies, and affective disorders scales of the YSR as clustering variables. Such scales were selected because, on the basis of the results that emerged in previous studies [30,44], we hypothesized that the presence of interpersonal–social difficulties and anxious–depressive symptoms may discriminate between groups with recent and past onset of NSSI. Cluster analyses were conducted on 94 of the 118 NSSI patients because of the missing data in the scales considered (i.e., not all items were answered).

Since we had no prior knowledge of the optimal number of clusters, solutions with two to five clusters were tested. The best clustering solution was selected according to the Bayesian Information Criterion (BIC; [52]), which is one of the most well-known and successfully applied criteria to compare different clustering solutions and to determine which most closely fits the data [53]. The Akaike’s Information Criterion (AIC; [54]), R-squared (R^2^), and silhouette values were also taken into consideration.

Each cluster was then characterized with respect to size, mean age, sex, and onset of NSSI distributions. The differences among clusters in terms of the onset of NSSI (past vs. recent) were examined through the **χ**^2^ test. The clusters that were the most relevant in defining the clinical profile of the patients in the two groups were also described with regard to the average age of the onset of NSSI, the presence of suicidal ideation and suicide attempts, and the mean scores on the DESR scale of the YSR. This last scale was considered to deepen the emotional dysregulation aspects, which were found to play a crucial role in NSSI behavior [41].

Subsequently, a binomial logistic regression was conducted to clarify which of the above-mentioned variables discriminate between the groups according to the onset of NSSI. Thus, the scales for social problems, social competencies, and affective disorders on the YSR were input as predictors, while the onset of NSSI was used as the dichotomous dependent variable.

Concerning the second step (i.e., to identify the potential risk factors for maintaining NSSI over time), **χ**^2^ tests were computed between the onset of NSSI and bullying, school problems, family problems, psychiatric familiarity, alcohol and substance use, traumatic life events, and borderline intrapsychic functioning.

The statistical significance level was defined as *p* < 0.05. The above-described analyses were performed using the JASP version 0.16.1 statistical software [55].

## 3. Results

### 3.1. Sociodemographic and Clinical Characteristics of Patients according to the NSSI Onset

The sample was composed of 38 inpatients with a past onset of NSSI (M_age_ = 14.7 years, SD = 1.63; average age of the onset of NSSI = 12.7 years, SD = 1.32) and 61 inpatients with a recent onset of NSSI (M_age_ = 14.2 years, SD = 1.34; average age of the onset of NSSI = 13.3 years, SD = 1.39). Because of the retrospective nature of the study, the data about the onset of NSSI were missing for 19 inpatients. The two groups did not significantly differ for age (t_97_ = −1.65, *p* = 0.10) and sex (**χ**^2^ (1) = 0.78, *p* = 0.38).

As regards ICD-10 diagnosis, 80.3% of inpatients in the recent NSSI onset group and 57.9% of the inpatients in the past NSSI onset group presented an internalizing disorder (e.g., F30–39 and F40–49 codes), while 15.8% of the inpatients in the first group and none of inpatients in the second group suffered from an externalizing disorder (e.g., F90–92 codes). Moreover, 19.7% of those with a recent NSSI onset and 26.3% of those with a past onset obtained an ICD-10 diagnosis of mixed disorder (i.e., with both internalizing and externalizing aspects), while 57.4% of the patients in the recent NSSI onset group and 60.5% in the past NSSI onset group had at least one comorbidity.

In both groups, the first main reason for hospitalization was related to suicidal phenomena (45.9% in the recent NSSI onset group and 39.4% in the past NSSI onset group), followed by NSSI as the second main reason (24.5% in the recent NSSI onset group and 26.3% in the past NSSI onset group). Hospitalization mainly occurred through emergency department admission (80.3% in the recent NSSI onset group and 63.1% in the past NSSI onset). In most cases, post-discharge services included local mental health services (50.8% in the recent NSSI onset group and 47.3% in the past NSSI onset group). The results for the other sociodemographic and clinical variables are presented in Table 1.

### 3.2. The Cluster Solution

In order to identify the optimal number of clusters, solutions with two to five clusters were tested and compared. As can be seen in Table 2, the solution with four clusters had the best BIC value and the other fit indices were all good; therefore, the four-cluster solution was selected because, overall, it turned out to be the most suitable for our data.

Four different psycho-behavioral profiles emerged from the final clustering solution. The first cluster, labeled “Moderate affective difficulties with discrepant social functioning”, was composed of 26 patients (23 girls and 3 boys; 88.5% and 11.5%, respectively) with a mean age of 14.8 years (SD = 1.4). The second cluster, named “Socio-affective impairment”, counted 21 patients (20 girls and 1 boy; 95.2% and 4.8%, respectively) with a mean age of 14.6 years (SD = 1.3). The third cluster, called “Good socio-affective functioning” included 24 patients (19 girls and 4 boys, 79.2% and 20.8%, respectively) with a mean age of 14.3 years (SD = 1.3). Finally, the fourth cluster, labeled “Low affective difficulties with discrepant social functioning”, consisted of 23 patients (19 girls and 4 boys; 82.6% and 17.4%, respectively) with a mean age of 14.6 years (SD = 1.3).

**Figure 1:**
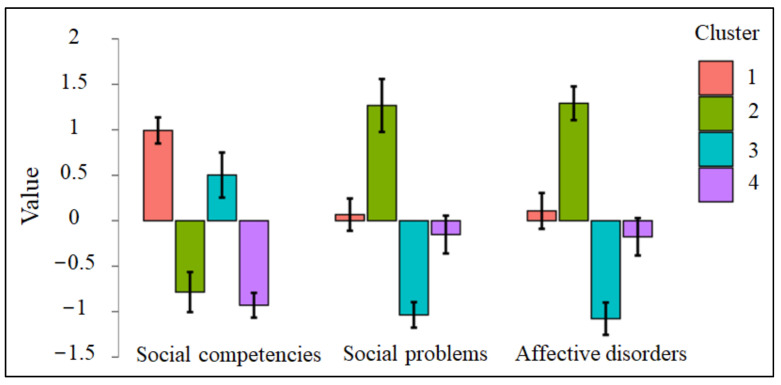
Trends of the four clusters on the selected YSR scales. *Notes*: Cluster 1 = moderate affective difficulties with discrepant social functioning; Cluster 2 = socio-affective impairment; Cluster 3 = good socio-affective functioning; Cluster 4 = low affective difficulties with discrepant social functioning.

As shown in Figure 1, each cluster presented a peculiar trend on the selected YSR scales, with an opposite trend between the first and fourth clusters and between the second and third clusters. More specifically, the first cluster was characterized by some affective difficulties, while a mixed social profile emerged because patients presented good social competencies in conjunction with social problems. The fourth cluster was characterized by a better affective functioning, but, in a similar but different way to the previous cluster, the social profile was not clearly defined, given that patients had low social problems concurrently with poor social competencies. Regarding the second cluster, it was featured by both social and affective difficulties, since patients reported scarce social competencies coexisting with high affective disorders and social problems. On the contrary, the third cluster was characterized by a good socio-affective functioning deriving from high social competencies and low social and affective difficulties.

Concerning the number of patients with past and recent onset of NSSI in each cluster, in the “Moderate affective difficulties with discrepant social functioning” cluster, 9 patients (40.9%) reported recent onset of NSSI, while 13 (59.1%) reported past onset; in the “Socio-affective impairment” cluster, 13 patients (76.5%) presented recent onset of NSSI, while 4 (23.5%) reported past onset; in the “Good socio-affective functioning” cluster, 12 patients (54.5%) had recent onset of NSSI and 10 (45.5%) had past onset; finally, in the “Low affective difficulties with discrepant social functioning” cluster, 17 patients (89.5%) reported recent onset of NSSI, while 2 (10.5%) reported past onset. The chi-square test showed that the between-cluster difference with respect to the onset of NSSI was statistically significant (**χ**^2^ (3) = 12.40, *p* = 0.006), thus indicating that the inclusion in one of the clusters was not independent of recent or past NSSI onset. In particular, as can be noticed above, patients of the two groups were almost equally present in the “Moderate affective difficulties with discrepant social functioning” and “Good socio-affective functioning” clusters, while a significantly greater number of patients with recent NSSI onset was observed in the “Socio-affective impairment” and “Low affective difficulties with discrepant social functioning” clusters. Therefore, these last clusters were considered the most relevant in discriminating between the two groups of nonsuicidal self-harming patients and in defining their psycho-behavioral profile. Specifically, since said clusters were both characterized by low social competencies, it is reasonable to assume that a lack of social skills may be particularly involved in a recent onset of NSSI, thus playing an important role in differentiating these patients from those with past onset.

As regards the other clinical characteristics of these two clusters, the average age of the NSSI onset was 12.9 years (SD = 1.19) in the “Socio-affective impairment” cluster. Moreover, all patients presented suicidal ideation, and 9 (42.9%) attempted suicide. Finally, the mean DESR scale score was 219.3 (SD = 21.9) on the YSR; given that a DESR score between 180 and 210 indicates an impairment in emotional regulation function and a score of ≥210 indicates a severe dysregulation, this cluster was composed of patients with severe emotional dysregulation. Concerning the “Low affective difficulties with discrepant social functioning” cluster, the average age of NSSI onset was 13.7 years (SD = 1.2). In this cluster, 19 patients (82.6%) reported suicidal ideation and 8 (34.8%) attempted suicide. Finally, the mean DESR scale score was 183.1 (SD = 17.5) on the YSR; therefore, the patients in this cluster were characterized by a moderate impairment in emotional regulation function.

Then, a binomial logistic regression was conducted using the onset of NSSI as the dependent variable and the social competencies, social problems, and affective disorders scales of the YSR as predictors. This analysis was run to determine which variables discriminate between the two groups of patients (i.e., with past and recent onset of NSSI). The model resulting from the analysis explained between 19% (McFadden’s R^2^) and 30% (Nagelkerke’s R^2^) of the variance (**χ**^2^ (3) = 19.5, *p* < 0.001), thus successfully distinguishing patients with past NSSI onset from those with recent onset. As can be seen in Table 3, the significant predictors emerged as just the YSR scales for social competencies and affective disorders. To be specific, higher scores for both scales were significantly more likely in the group with past NSSI onset, suggesting that these patients may have developed good social skills in the face of affective difficulties. Consequently, the binomial logistic regression confirmed the results that previously emerged from the cluster analysis, namely, the pivotal role played by social competencies in discriminating between the two groups of patients. Moreover, this analysis indicated that affective problems should also be carefully taken into consideration, particularly as a potential risk factor for the maintenance of nonsuicidal self-harming behavior over time.

### 3.3. Risk Factors for the Maintenance of NSSI over Time

To determine the personal and clinical variables implicated in the maintenance of NSSI over time, several chi-square tests were conducted. No statistically significant results emerged considering the onset of NSSI and bullying (χ^2^ (1) = 1.02, *p* = 0.31), family problems (χ^2^ (1) = 0.46, *p* = 0.49), traumatic life events (χ^2^ (1) = 0.43, *p* = 0.52), borderline personality organization (χ^2^ (1) = 1.62, *p* = 0.20), and psychiatric familiarity (χ^2^ (1) = 0.24, *p* = 0.63). On the contrary, significant relationships were found between the onset of NSSI and alcohol use (χ^2^ (1) = 4.74, *p* = 0.03), substance use (χ^2^ (1) = 7.05, *p* = 0.008), and school problems (χ^2^ (1) = 5.10, *p* = 0.02). Specifically, patients with past NSSI onset had a higher probability of using alcohol (34.2%) and substances (34.2%) and reporting school problems (83.8%) compared to patients with recent onset (probability of 15.3%, 11.9%, and 62.3% respectively). Therefore, the presence of school problems and alcohol and substance (over)consumption may constitute critical factors dictating the continuation of nonsuicidal self-harming behavior over time.

## 4. Discussion

The increased frequency of NSSI in adolescents has been widely documented in the literature [7,13,56,57]; nonetheless, only a few studies have focused on patients’ characteristics on the basis of NSSI course in terms of temporal duration of self-injurious acts. Therefore, the present study aimed to fill this gap in the literature, considering a population of young Italian inpatients who presented with a history of NSSI.

The first specific aim was to define the psycho-behavioral profile of patients, evaluating whether belonging to the group with past NSSI onset (i.e., NSSI for more than one year at the time of hospitalization) or recent (i.e., NSSI for less than one year) was associated with a different severity in terms of clinical symptomatology. From the cluster analysis, four different profiles emerged, each of them characterized by a peculiar trend on the considered variables (i.e., social competences, social problems, and affective disorders). This result seems to confirm that NSSI is a complex phenomenon, common to different social and affective profiles, and not always associated with the presence of a structured psychiatric disorder [58].

In specific reference to the objective of this study, the principal clusters that emerged were “Socio-affective impairment” and “Low affective difficulties with discrepant social functioning”; the former was characterized by difficulties in social and affective functioning, while the latter was characterized by good affective functioning and an undefined social profile, since low social problems and low social competences were both present. In both clusters, more than half of inpatients had a recent onset of NSSI, and the common feature of the two profiles was the presence of low social competences; these thereby seem to have a central role in discriminating between patients with recent and past NSSI onset. This result, on the one hand, is in line with research highlighting the importance of social factors in the onset of NSSI [41,59] and, on the other hand, adds that a lack of social skills, rather than the presence of social problems, may be more implicated in the onset of NSSI and its short-term maintenance.

Both the above-mentioned clusters were characterized not only by low social competences, but also by severe difficulties in emotional regulation, as indicated by the mean scores on the DESR scale of the YSR. These data support the hypothesis that the social competencies dimension, which also includes the ability to understand, interpret, and use social interaction rules, may be linked to the ability to regulate and express emotions. Regarding this topic, an important construct is alexithymia, a well-known risk factor for NSSI and other self-injurious behaviors in adolescents and young adults [60,61,62]. For example, a recent work reported an association between alexithymia and social–cognitive abilities; in particular, alexithymia was significatively linked to difficulties in the recognition of other people’s expressions, emotional regulation, and empathy [63]. The authors stated that the ability to identify and correctly recognize one’s own emotions is essential for the recognition of other people’s emotions and feelings and, as a consequence, for social relationships. Therefore, it is possible to hypothesize that deficits in emotional regulation may limit an adolescent’s ability to understand another person’s emotional status, thus also compromising social abilities. In this scenario, and considering the importance of peer relationships in adolescence, acting out self-injurious behaviors may have the purpose of reducing inner tension and overcoming interpersonal distress due to an adolescent’s difficulties in establishing his own identity and social role [64]. Furthermore, NSSI may constitute a way of being accepted and included among peers in the face of poor social competences and an inability to identify alternative strategies [65]. In light of the relationship between alexithymia and the development of social abilities, and the role of both these factors in the onset of NSSI, longitudinal studies that consider both social and emotional competences should be performed in order to better investigate the course of NSSI in teenagers.

The “Socio-affective impairment” cluster, characterized by a significant social and affective impairment, was more frequently associated with suicidality than the “Low affective difficulties with discrepant social functioning” cluster. This is in line with the literature, showing that the presence of affective disorders and NSSI represents a possible risk factor for more severe forms of self-injurious behaviors [66,67]. In addition, the concomitant presence of social difficulties and, thus, the lack of an adequate social support network may contribute to worsening the clinical picture, resulting in a greater risk of acting out. These data underline the importance of evaluating social functioning in patients with NSSI. Particularly, such a result may be useful in the early identification of youth at risk of suicide, as stated in several works that displayed the pivotal role of social competences in the evolution from less severe forms of self-injury to the enactment of suicidal behaviors [39,68,69].

Another noteworthy point regards the clusters characterized by a discrepant social functioning—namely, “Moderate affective difficulties with discrepant social functioning” and “Low affective difficulties with discrepant social functioning.” The features of such clusters showed that the presence of good social competences in patients with NSSI does not necessarily exclude the presence of concomitant social problems, nor does the presence of scarce social competences imply the co-occurrence of social problems. This result, on the one hand, emphasizes the complex social functioning of patients with NSSI and, on the other hand, points out that social competences and social problems may be independent constructs, at least in the NSSI population and as assessed by the YSR. However, future studies are warranted in order to better deepen this aspect.

Taken together, our findings show the importance of focusing on social competences for a better understanding of NSSI in adolescence and to prevent its onset. In this sense, ad hoc interventions are needed with the aim of not only reducing social difficulties, but also fostering the development of adequate social competences and emotional regulation strategies that enable adolescents to face intra-psychical conflicts and relational problems typical of this age.

Subsequently, the logistic binomial regression confirmed and broadened the results that emerged from the cluster analysis. Specifically, it demonstrated that the scores on the YSR scales pertaining to social competences and affective disorders were significant predictors of belonging to one of the two groups (i.e., past or recent NSSI onset). According to the psycho-behavioral profiles previously delineated, inpatients in the recent NSSI onset group were more likely to present low social competences. The presence of affective problems, in contrast, were more probable in the group with past onset; therefore, these problems may play a role in predicting the maintenance of NSSI over time, in line with our hypothesis and the literature [30,41]. For some authors, the presence of a mood disorder could be considered itself a predictor of NSSI [70,71,72], and the association between depression and NSSI is strong [73]. According to the “Pain Offset Relief” [74] and “Experiential Avoidance Model” [33] theories, acting out NSSI enables one to temporarily avoid negative emotional experiences; however, the intensity of the underlying emotions, not regulated by more adaptive modalities, could become more difficult to face over time, resulting in more severe affective disorders. Therefore, adolescents with mood deflection would use self-injurious acts as a coping strategy to seek relief from depressive symptoms [75,76,77]. In this context, we can hypothesize that the presence of mood disorders could facilitate the maintenance of NSSI over time.

Concerning the second objective of the study—that is, the definition of possible risk factors for the maintenance of NSSI over time—a significant relationship between the belonging group and the presence of school problems and use of alcohol and substances emerged. In particular, patients with past NSSI onset were more likely to report school problems and present abuse of alcohol and substances compared to patients in the recent onset group. On the contrary, some personal and clinical characteristics considered by the literature as risk factors for NSSI, namely, family problems [21], psychiatric familiarity [14], bullying [18], traumatic events [17], and borderline personality functioning [15], were not associated with the belonging group. Our results suggest that these variables may be transversally associated with NSSI, thus not being distinctive factor, either for the onset or the maintenance of self-injurious behaviors. Alcohol and substance abuse and school problems may instead pose a risk for the maintenance of NSSI over time; therefore, young self-injuring patients who present with these problems may be more at risk of chronic NSSI.

The presence of school problems could be associated with stress and anxiety related to school performance, and these negative affective states could be particular risk factors not only for the onset [65]**,** but also for the maintenance of NSSI. In addition, it is important to consider the role of psychiatric disorders in the relationship between school problems and NSSI. In fact, on the one hand, school problems may increase the risk of developing psychopathologies, of which NSSI could be the manifestation [78]; on the other hand, they could derive from pre-existent psychological disorders to which NSSI could be linked, thus contributing to worsening of the clinical picture and facilitating the maintenance of self-injurious acts. However, the relationship between school problems and the maintenance of NSSI should be better investigated in future studies to clarify how psychiatric disorders intervene in such a relationship.

Regarding alcohol and substance use/abuse, a longitudinal study on adolescents [79] showed a gradual reduction in self-injurious acts over time but, on the other hand, increased substance use. This seems to suggest a “shift” in NSSI symptoms toward a harmful and, in some respects, more socially accepted behavior [79]. Therefore, we can hypothesize that the presence of substance use/abuse in adolescents with long-lasting NSSI may represent the behavioral evolution of the difficulties in emotional regulation, impulsivity, emotional lability, and inadequate coping strategies associated with both self-injurious acts and substance dependence. Finally, some authors have hypothesized an indirect causal model, according to which the abuse of substances, by causing an impairment of judgment abilities, pain reduction, or fantasy stimulation, may induce or trigger NSSI [80,81].

Despite the intriguing results that emerged from our study, some limitations need to be mentioned, such as the study design (transversal and retrospective), the limited sample size, and the heterogeneity of the population, which may not be representative of the general adolescent population with NSSI. In particular, the small sample size did not enable us to perform a discriminant validation analysis of the clusters; future studies involving larger groups of adolescents with NSSI are thereby needed to further confirm the validity of the clusters that emerged in the present study. Moreover, the use of a self-report test, particularly when investigating perceived social skills and emotional difficulties, may not be an objective assessment of interpersonal skills, since the responses of participants may be influenced by individual biases, social desirability, or failure to understand a question. Taking a multi-method and multi-informant perspective, qualitative studies are needed to better understand the nature of NSSI and the factors associated with it; in fact, in the context of anamnestic collection and clinical interviews with patients and caregivers, it may be particularly useful to also consider information that is not immediately provided by standardized self-report tests. Finally, longitudinal studies are encouraged in order to deepen the factors associated with recent or past NSSI onset and the predictors of the maintenance of this behavior, as well as of the evolution toward more severe types of self-injury.

## 5. Conclusions

The main aim of the present study was to explore the psycho-behavioral profiles of patients with NSSI according to the time of the onset of self-injurious acts. Our results indicated that low social competences, probably related to difficulties in emotional regulation, may be involved in the recent onset of NSSI. Therefore, these patients could use NSSI as a strategy to cope with internal tensions, be accepted by peers, and build their own identity in the face of the inability to find more adaptive strategies. Patients with past onset of NSSI, despite having better social competences, presented higher affective difficulties. Consequently, affective disorders could facilitate a chronic course of NSSI that, in this context, would represent a stable behavioral strategy for finding relief from negative emotional states. Other risk factors for the maintenance of NSSI over time emerged as use/abuse of alcohol and substances and school problems. In particular, we can hypothesize that the relationship between school problems and long-lasting NSSI is affected by psychopathological disorders, but further studies are needed to better define this aspect. Regarding alcohol and substance abuse, impulsivity, emotional dysregulation, and inadequate coping strategies may be the mechanisms underlying the behavioral evolution of NSSI to a more socially accepted behavior, thus contributing to the maintenance of NSSI over time.

Finally, our results underline the importance of evaluating social competences, strictly related to alexithymia, in young patients with NSSI.

Altogether, these findings may have important implications for clinical practice and in primary and secondary prevention, particularly considering NSSI as one of the main risk factors for suicidal behaviors. It is therefore necessary to increase awareness of NSSI as a risk factor and deal with the aspects associated with it in both medical personnel and family and professional figures involved in the adolescent’s growth process. To this end, it is fundamental to pay attention to children and adolescents with low social competences, emotional difficulties, and behavioral problems (such as school difficulties and substance use) in order to encourage early detection of vulnerabilities and offer interventions regarding socialization and emotional–behavioral regulation. Likewise, it is paramount to plan appropriate multidisciplinary care for young people with self-harming behavior to ensure close monitoring and treatment of possible comorbidity, thus also preventing the risk of evolution over time towards more serious acts, such as suicidal behaviors.

## Figures and Tables

**Table 1:** Sociodemographic and clinical features of patients with past and recent NSSI onset.

	Past NSSI Onset %	Recent NSSI Onset %
Sociodemographic variables		
Female	84.21	90.16
Caucasian	89.47	88.52
Immigration	18.42	21.31
Separated parents	28.94	28.33
Only child	23.68	16.39
High school educational level	65.78	59.01
Maternal unemployment	10.52	14.75
Paternal unemployment	5.26	3.27
Individual variables		
School problems	83.78	62.29
Bullying/cyberbullying	37.83	48.33
Social withdrawal	10.81	21.31
Conflictual peer relationships	35.13	36.06
Intensive use of online social networks	56.52	53.19
Alcohol use/abuse	34.21	15.25
Substance use/abuse	34.21	11.86
Smoking	47.36	16.94
Borderline functioning	58.33	44.82
Traumatic life events	52.63	45.90
Eating disorders	10.52	14.75
Chronic pathology	23.68	37.70
Family variables		
Family health problems	51.35	45.90
Intra-family problems	73.68	67.21
Psychiatric familiarity	67.64	72.41
Variables related to admission to mental health services		
Previous hospitalizations	39.47	29.50
1-year post-discharge relapse	10.52	31.14
Previous accesses in other mental health services	89.47	75.40
Pharmacological therapy	89.18	83.33
Psychotherapy	94.28	89.65
Self-injurious phenomena variables		
Attempted suicide	39.47	40.98
Suicidal ideation	81.57	85.24
High NSSI frequency	63.15	45.90
Multiple self-injured body parts	75.75	56.36
Intrapersonal function of NSSI	100	93.47

**Table 2:** Fit indices of solutions with two, three, four, and five clusters.

Clustering Solution	R^2^	AIC	BIC	Silhouette
Two clusters	0.41	174.29	189.55	0.35
Three clusters	0.58	134.04	157.93	0.34
Four clusters	0.68	114.36	144.88	0.34
Five clusters	0.72	109.13	147.28	0.31

Note. AIC = Akaike’s Information Criterion; BIC = Bayesian Information Criterion.

**Table 3:** Results of the binomial logistic regression considering the NSSI onset (past vs. recent) as a dependent variable.

YSR Scales	β	*Z*	*p*	χ^2^ (df)	Odds Ratio (95% CI)
Social competencies	0.11	3.26	0.001	13.05 (1)	1.11 (1.04–1.19)
Social problems	−0.07	−1.75	0.08	3.44 (1)	0.93 (0.86–1.01)
Affective disorders	0.09	2.81	0.005	9.47 (1)	1.09 (1.02–1.16)

## Data Availability

The data that support the findings of this study are available from the corresponding author, S.I., upon reasonable request.

## References

[1] Nock M., Joinerjr T., Gordon K., Lloydrichardson E., Prinstein M. (2006). Non-Suicidal Self-Injury among Adolescents: Diagnostic Correlates and Relation to Suicide Attempts. Psychiatry Res..

[2] Nock M., Favazza A.R. (2009). Non-Suicidal Self-Injury: Definition and Classification. Understanding Non-Suicidal Self-Injury: Origins, Assessment, and Treatment.

[3] Nock M.K. (2010). Self-Injury. Annu. Rev. Clin. Psychol..

[4] Plener P.L., Fegert J.M., Kaess M., Kapusta N.D., Brunner R., Groschwitz R.C., In-Albon T., Resch F., Becker K. (2017). Nonsuicidal Self-Injury in Adolescence: A Clinical Guideline for Diagnostics and Therapy. Z. Kinder Jugendpsychiatr. Psychother..

[5] Baetens I., Claes L., Muehlenkamp J., Grietens H., Onghena P. (2011). Non-Suicidal and Suicidal Self-Injurious Behavior among Flemish Adolescents: A Web-Survey. Arch. Suicide Res..

[6] Zanus C., Battistutta S., Aliverti R., Montico M., Cremaschi S., Ronfani L., Monasta L., Carrozzi M. (2017). Adolescent Admissions to Emergency Departments for Self-Injurious Thoughts and Behaviors. PLoS ONE.

[7] Cipriano A., Cella S., Cotrufo P. (2017). Nonsuicidal Self-Injury: A Systematic Review. Front. Psychol..

[8] Kaess M., Parzer P., Mattern M., Plener P.L., Bifulco A., Resch F., Brunner R. (2013). Adverse Childhood Experiences and Their Impact on Frequency, Severity, and the Individual Function of Nonsuicidal Self-Injury in Youth. Psychiatry Res..

[9] Wolff J., Frazier E.A., Esposito-Smythers C., Burke T., Sloan E., Spirito A. (2013). Cognitive and Social Factors Associated with NSSI and Suicide Attempts in Psychiatrically Hospitalized Adolescents. J. Abnorm. Child Psychol..

[10] Cerutti R., Manca M., Presaghi F., Gratz K.L. (2011). Prevalence and Clinical Correlates of Deliberate Self-Harm among a Community Sample of Italian Adolescents. J. Adolesc..

[11] Giletta M., Scholte R.H.J., Engels R.C.M.E., Ciairano S., Prinstein M.J. (2012). Adolescent Non-Suicidal Self-Injury: A Cross-National Study of Community Samples from Italy, the Netherlands and the United States. Psychiatry Res..

[12] Gatta M., Rago A., Dal Santo F., Spoto A., Battistella P.A. (2016). Non-Suicidal Self-Injury among Northern Italian High School Students: Emotional, Interpersonal and Psychopathological Correlates. J. Psychopathol..

[13] Zetterqvist M., Jonsson L.S., Landberg Å., Svedin C.G. (2021). A Potential Increase in Adolescent Nonsuicidal Self-Injury during COVID-19: A Comparison of Data from Three Different Time Points during 2011–2021. Psychiatry Res..

[14] Arbuthnott A.E., Lewis S.P. (2015). Parents of Youth Who Self-Injure: A Review of the Literature and Implications for Mental Health Professionals. Child Adolesc. Psychiatry Ment. Health.

[15] Benzi I.M.A., Sarno I., Di Pierro R. (2018). Maladaptive Personality Functioning and Non-Suicidal Self Injury in Adolescence. Clin. Neuropsychiatry.

[16] Fischer-Kern M., Buchheim A., Hörz S., Schuster P., Doering S., Kapusta N.D., Taubner S., Tmej A., Rentrop M., Buchheim P. (2010). The Relationship between Personality Organization, Reflective Functioning, and Psychiatric Classification in Borderline Personality Disorder. Psychoanal. Psychol..

[17] Serafini G., Canepa G., Adavastro G., Nebbia J., Belvederi Murri M., Erbuto D., Pocai B., Fiorillo A., Pompili M., Flouri E. (2017). The Relationship between Childhood Maltreatment and Non-Suicidal Self-Injury: A Systematic Review. Front. Psychiatry.

[18] Brunstein Klomek A., Snir A., Apter A., Carli V., Wasserman C., Hadlaczky G., Hoven C.W., Sarchiapone M., Balazs J., Bobes J. (2016). Association between Victimization by Bullying and Direct Self Injurious Behavior among Adolescence in Europe: A Ten-Country Study. Eur. Child Adolesc. Psychiatry.

[19] Baetens I., Greene D., Van Hove L., Van Leeuwen K., Wiersema J.R., Desoete A., Roelants M. (2021). Predictors and Consequences of Non-suicidal Self-injury in Relation to Life, Peer, and School Factors. J. Adolesc..

[20] Jarvi S.M., Swenson L.P. (2017). The Role of Positive Expectancies in Risk Behavior: An Exploration of Alcohol Use and Nonsuicidal Self-Injury. Crisis J. Crisis Interv. Suicide Prev..

[21] Victor S.E., Hipwell A.E., Stepp S.D., Scott L.N. (2019). Parent and Peer Relationships as Longitudinal Predictors of Adolescent Non-Suicidal Self-Injury Onset. Child Adolesc. Psychiatry Ment. Health.

[22] Gatta M., Sisti M., Sudati L., Miscioscia M., Simonelli A. (2016). The Lausanne Trilogue Play within the Outcome Evaluation in Infant Mental Health: A Preliminary Report. Res. Psychother. Psychopathol. Process Outcome.

[23] Gatta M., Miscioscia M., Sisti M., Comis I., Battistella P.A. (2017). Interactive Family Dynamics and Non-Suicidal Self-Injury in Psychiatric Adolescent Patients: A Single Case Study. Front. Psychol..

[24] You J., Leung F. (2012). The Role of Depressive Symptoms, Family Invalidation and Behavioral Impulsivity in the Occurrence and Repetition of Non-Suicidal Self-Injury in Chinese Adolescents: A 2-Year Follow-up Study. J. Adolesc..

[25] Wang B., You J., Lin M.-P., Xu S., Leung F. (2016). Developmental Trajectories of Nonsuicidal Self-Injury in Adolescence and Intrapersonal/Interpersonal Risk Factors. J. Res. Adolesc..

[26] You J., Leung F., Fu K., Lai C.M. (2011). The Prevalence of Nonsuicidal Self-Injury and Different Subgroups of Self-Injurers in Chinese Adolescents. Arch. Suicide Res..

[27] Hankin B.L., Abela J.R.Z. (2011). Nonsuicidal Self-Injury in Adolescence: Prospective Rates and Risk Factors in a 2 ½year Longitudinal Study. Psychiatry Res..

[28] Liu R.T. (2017). Characterizing the Course of Non-Suicidal Self-Injury: A Cognitive Neuroscience Perspective. Neurosci. Biobehav. Rev..

[29] Selby E.A., Kranzler A., Fehling K.B., Panza E. (2015). Nonsuicidal Self-Injury Disorder: The Path to Diagnostic Validity and Final Obstacles. Clin. Psychol. Rev..

[30] Victor S.E., Styer D., Washburn J.J. (2016). Functions of Nonsuicidal Self-Injury (NSSI): Cross-Sectional Associations with NSSI Duration and Longitudinal Changes over Time and Following Treatment. Psychiatry Res..

[31] Fox K.R., Franklin J.C., Ribeiro J.D., Kleiman E.M., Bentley K.H., Nock M.K. (2015). Meta-Analysis of Risk Factors for Nonsuicidal Self-Injury. Clin. Psychol. Rev..

[32] Glenn C.R., Klonsky E.D. (2011). Prospective Prediction of Nonsuicidal Self-Injury: A 1-Year Longitudinal Study in Young Adults. Behav. Ther..

[33] Chapman A.L., Gratz K.L., Brown M.Z. (2006). Solving the Puzzle of Deliberate Self-Harm: The Experiential Avoidance Model. Behav. Res. Ther..

[34] Nock M.K., Prinstein M.J. (2004). A Functional Approach to the Assessment of Self-Mutilative Behavior. J. Consult. Clin. Psychol..

[35] Andrews T., Martin G., Hasking P., Page A. (2013). Predictors of Continuation and Cessation of Nonsuicidal Self-Injury. J. Adolesc. Health.

[36] Kiekens G., Hasking P., Bruffaerts R., Claes L., Baetens I., Boyes M., Mortier P., Demyttenaere K., Whitlock J. (2017). What Predicts Ongoing Nonsuicidal Self-Injury?: A Comparison Between Persistent and Ceased Self-Injury in Emerging Adults. J. Nerv. Ment. Dis..

[37] Lockwood J., Townsend E., Daley D., Sayal K. (2020). Impulsivity as a Predictor of Self-Harm Onset and Maintenance in Young Adolescents: A Longitudinal Prospective Study. J. Affect. Disord..

[38] Asarnow J.R., Porta G., Spirito A., Emslie G., Clarke G., Wagner K.D., Vitiello B., Keller M., Birmaher B., McCracken J. (2011). Suicide Attempts and Nonsuicidal Self-Injury in the Treatment of Resistant Depression in Adolescents: Findings from the TORDIA Study. Adolesc Psychiatry.

[39] Hamza C.A., Stewart S.L., Willoughby T. (2012). Examining the Link between Nonsuicidal Self-Injury and Suicidal Behavior: A Review of the Literature and an Integrated Model. Clin. Psychol. Rev..

[40] Jacobson C.M., Muehlenkamp J.J., Miller A.L., Turner J.B. (2008). Psychiatric Impairment Among Adolescents Engaging in Different Types of Deliberate Self-Harm. J. Clin. Child Adolesc. Psychol..

[41] Muehlenkamp J., Brausch A., Quigley K., Whitlock J. (2013). Interpersonal Features and Functions of Nonsuicidal Self-Injury. Suicide Life Threat. Behav..

[42] Nock M.K. (2009). Why Do People Hurt Themselves?: New Insights Into the Nature and Functions of Self-Injury. Curr. Dir. Psychol. Sci..

[43] Edmondson A.J., Brennan C.A., House A.O. (2016). Non-Suicidal Reasons for Self-Harm: A Systematic Review of Self-Reported Accounts. J. Affect. Disord..

[44] Emery A.A., Heath N.L., Mills D.J. (2017). The Role of Basic Need Satisfaction in the Onset, Maintenance, and Cessation of Non-Suicidal Self-Injury: An Application of Self-Determination Theory. Arch. Suicide Res..

[45] World Health Organization (1993). The ICD-10 Classification of Mental and Behavioural Disorders.

[46] Kernberg O.F. (1987). Disturbi Gravi Della Personalità. Bollati Boringhieri.

[47] Achenbach T.M., Rescorla L. (2001). Manual for the ASEBA Preschool Forms & Profiles: An Integrated System of Multi-Informant Assessment.

[48] Frigerio A., Cattaneo C., Cataldo M., Schiatti A., Molteni M., Battaglia M. (2004). Behavioral and Emotional Problems Among Italian Children and Adolescents Aged 4 to 18 Years as Reported by Parents and Teachers. Eur. J. Psychol. Assess..

[49] Frigerio A., Vanzin L., Pastore V., Nobile M., Giorda R., Marino C., Molteni M., Rucci P., Ammaniti M., Lucarelli L. (2006). The Italian Preadolescent Mental Health Project (PrISMA): Rationale and Methods. Int. J. Methods Psychiatr. Res..

[50] Biederman J., Petty C.R., Day H., Goldin R.L., Spencer T., Faraone S.V., Surman C.B.H., Wozniak J. (2012). Severity of the Aggression/Anxiety-Depression/Attention Child Behavior Checklist Profile Discriminates Between Different Levels of Deficits in Emotional Regulation in Youth With Attention-Deficit Hyperactivity Disorder. J. Dev. Behav. Pediatr..

[51] Biederman J., Petty C.R., Monuteaux M.C., Evans M., Parcell T., Faraone S.V., Wozniak J. (2009). The Child Behavior Checklist-Pediatric Bipolar Disorder Profile Predicts a Subsequent Diagnosis of Bipolar Disorder and Associated Impairments in ADHD Youth Growing Up: A Longitudinal Analysis. J. Clin. Psychiatry.

[52] Schwarz G. (1978). Estimating the Dimension of a Model. Ann. Stat..

[53] Fraley C. (1998). How Many Clusters? Which Clustering Method? Answers Via Model-Based Cluster Analysis. Comput. J..

[54] Akaike H. (1973). Information Theory and an Extension of the Maximum Likelihood Principle. Proceedings of the Second International Symposium on Information Theory.

[55] JASP Team JASP (Version 0.16.1). https://jasp-stats.org/previous-versions/.

[56] Hawton K., Hall S., Simkin S., Bale L., Bond A., Codd S., Stewart A. (2003). Deliberate Self-Harm in Adolescents: A Study of Characteristics and Trends in Oxford, 1990–2000: Deliberate Self-Harm in Adolescents. J. Child Psychol. Psychiatry.

[57] Nock M.K. (2008). Actions Speak Louder than Words: An Elaborated Theoretical Model of the Social Functions of Self-Injury and Other Harmful Behaviors. Appl. Prev. Psychol..

[58] Cassels M., Wilkinson P. (2016). Non-Suicidal Self-Injury in Adolescence. Paediatr. Child Health.

[59] Tatnell R., Kelada L., Hasking P., Martin G. (2014). Longitudinal Analysis of Adolescent NSSI: The Role of Intrapersonal and Interpersonal Factors. J. Abnorm. Child Psychol..

[60] Parolin M., Miscioscia M., De Carli P., Cristofalo P., Gatta M., Simonelli A. (2018). Alexithymia in Young Adults With Substance Use Disorders: Critical Issues About Specificity and Treatment Predictivity. Front. Psychol..

[61] Raffagnato A., Angelico C., Valentini P., Miscioscia M., Gatta M. (2020). Using the Body When There Are No Words for Feelings: Alexithymia and Somatization in Self-Harming Adolescents. Front. Psychiatry.

[62] Weissman D.G., Nook E.C., Dews A.A., Miller A.B., Lambert H.K., Sasse S.F., Somerville L.H., McLaughlin K.A. (2020). Low Emotional Awareness as a Transdiagnostic Mechanism Underlying Psychopathology in Adolescence. Clin. Psychol. Sci..

[63] Di Tella M., Adenzato M., Catmur C., Miti F., Castelli L., Ardito R.B. (2020). The Role of Alexithymia in Social Cognition: Evidence from a Non-Clinical Population. J. Affect. Disord..

[64] Heilbron N., Prinstein M.J. (2008). Peer Influence and Adolescent Nonsuicidal Self-Injury: A Theoretical Review of Mechanisms and Moderators. Appl. Prev. Psychol..

[65] Evans R., Hurrell C. (2016). The Role of Schools in Children and Young People’s Self-Harm and Suicide: Systematic Review and Meta-Ethnography of Qualitative Research. BMC Public Health.

[66] Victor S.E., Klonsky E.D. (2014). Correlates of Suicide Attempts among Self-Injurers: A Meta-Analysis. Clin. Psychol. Rev..

[67] Tuisku V., Kiviruusu O., Pelkonen M., Karlsson L., Strandholm T., Marttunen M. (2014). Depressed Adolescents as Young Adults –Predictors of Suicide Attempt and Non-Suicidal Self-Injury during an 8-Year Follow-Up. J. Affect. Disord..

[68] Klonsky E.D., May A.M. (2015). The Three-Step Theory (3ST): A New Theory of Suicide Rooted in the “Ideation-to-Action” Framework. Int. J. Cogn. Ther..

[69] Klonsky E.D., May A.M., Saffer B.Y. (2016). Suicide, Suicide Attempts, and Suicidal Ideation. Annu. Rev. Clin. Psychol..

[70] Lee W.K. (2016). Psychological Characteristics of Self-Harming Behavior in Korean Adolescents. Asian J. Psychiatry.

[71] Plener P.L., Schumacher T.S., Munz L.M., Groschwitz R.C. (2015). The Longitudinal Course of Non-Suicidal Self-Injury and Deliberate Self-Harm: A Systematic Review of the Literature. Bord. Personal. Disord. Emot. Dysregul..

[72] Rodav O., Levy S., Hamdan S. (2014). Clinical Characteristics and Functions of Non-Suicide Self-Injury in Youth. Eur. Psychiatry.

[73] Brunner R., Kaess M., Parzer P., Fischer G., Carli V., Hoven C.W., Wasserman C., Sarchiapone M., Resch F., Apter A. (2014). Life-Time Prevalence and Psychosocial Correlates of Adolescent Direct Self-Injurious Behavior: A Comparative Study of Findings in 11 European Countries. J. Child Psychol. Psychiatry.

[74] Franklin J.C., Puzia M.E., Lee K.M., Lee G.E., Hanna E.K., Spring V.L., Prinstein M.J. (2013). The Nature of Pain Offset Relief in Nonsuicidal Self-Injury: A Laboratory Study. Clin. Psychol. Sci..

[75] Andover M.S., Morris B.W. (2014). Expanding and Clarifying the Role of Emotion Regulation in Nonsuicidal Self-Injury. Can. J. Psychiatry.

[76] Kumar G., Pepe D., Steer R.A. (2004). Adolescent Psychiatric Inpatients’ Self-Reported Reasons for Cutting Themselves. J. Nerv. Ment. Dis..

[77] Muehlenkamp J.J., Gutierrez P.M. (2004). An Investigation of Differences Between Self-Injurious Behavior and Suicide Attempts in a Sample of Adolescents. Suicide Life. Threat. Behav..

[78] Epstein S., Roberts E., Sedgwick R., Polling C., Finning K., Ford T., Dutta R., Downs J. (2019). School Absenteeism as a Risk Factor for Self-Harm and Suicidal Ideation in Children and Adolescents: A Systematic Review and Meta-Analysis. Eur. Child Adolesc. Psychiatry.

[79] Nakar O., Brunner R., Schilling O., Chanen A., Fischer G., Parzer P., Carli V., Wasserman D., Sarchiapone M., Wasserman C. (2016). Developmental Trajectories of Self-Injurious Behavior, Suicidal Behavior and Substance Misuse and Their Association with Adolescent Borderline Personality Pathology. J. Affect. Disord..

[80] Moggi F. (2005). Etiological Theories on the Relationship of Mental Disorders and Substance Use Disorders. Dual Diagnosis: The Evolving Conceptual Framework.

[81] Sarno I., Madeddu F. (2007). Comportamenti Di Autoferimento, Uso Di Sostanze e Disturbo Borderline. Personalità/Dipendenze.

